# ﻿DNA barcoding of the leaf-miner flies (Diptera, Agromyzidae) of Mitaraka, French Guiana

**DOI:** 10.3897/zookeys.1083.76651

**Published:** 2022-01-25

**Authors:** Stéphanie Boucher, Jade Savage

**Affiliations:** 1 Department of Natural Resource Sciences, McGill University, Macdonald Campus, Ste-Anne-de-Bellevue, H9X 3V9, Quebec, Canada McGill University Quebec Canada; 2 Bishop’s University, Sherbrooke, J1M 1Z7, Quebec, Canada Bishop’s University Sherbrooke Canada

**Keywords:** Agromyzidae, Barcode Index Number (BIN), CO1, DNA barcoding, French Guiana, Neotropical

## Abstract

Species level identification of Agromyzidae based on morphology is often challenging due to their small size and morphological homogeneity. DNA barcoding has been used regularly to assist with the identification of economically important species of Agromyzidae, but rarely as a tool for species delineation or identification in biodiversity surveys. The main objective of this study was to investigate whether DNA barcoding and the BIN (Barcoding Index) system could assist with species identification, species delineation, male/ female association, and diversity assessment of Agromyzidae material previously determined to morphospecies from Mitaraka, French Guiana. Amplification success was low, with sequences over 400 bp recovered for only 24 (48%) of the selected specimens. Sequences assigned to 17 morphospecies formed 16 distinct branches or clusters separated by very high (minimum of 10%) sequence divergence. Following the reassessment and subsequent reassignment of one specimen, congruence between morphology and DNA barcodes was high with a single instance of two morphospecies sharing identical sequences. While DNA barcoding did not assist with identification (none of our sequences matched those of named taxa in BOLD or GenBank), it did provide support for most of our morphospecies concepts, including male/female associations. The BIN system also provided access to information about the distribution and habitat preferences of several taxa. We conclude that DNA barcoding was a useful approach to study the species diversity of our samples but that much work remains to be done before it can be used as an identification tool for the Agromyzidae fauna of Mitaraka and the rest of the Neotropical region.

## ﻿Introduction

The Agromyzidae is a family of small flies, measuring on average 2–4 mm in wing length, although they can be smaller than 1 mm or measure up to 6.5 mm. Their coloration is variable, from yellow and/or black, brown, or grey, sometimes with metallic greenish, bluish, or coppery coloration. Most have clear wings, but they may be patterned or infuscated in a few tropical species. The family contains approximately 3200 described species found worldwide (von Tschirnhaus 2021). The larvae of all species feed internally on living plant tissues, with most species with known biology developing inside leaves, hence their common name of leaf-miner flies. The family includes some important pest species of agricultural and ornamental plants, including three well known species occurring in many parts of the world, including South America: *Liriomyzahuidobrensis* (Blanchard), *Liriomyzatrifolii* (Burgess), *Liromyzasativae* Blanchard. Agromyzidae species identification based on morphology alone is a difficult task due to their small size and morphological homogeneity, but also due to their high diversity, presence of numerous undescribed species and lack of recent identification keys for many countries (Benavent-Corai et al. 2005; Boucher 2010; Boucher and Pollet 2021). Misidentification has happened repeatedly in the literature even when identification was performed by specialists (Scheffer and Winkler 2008). Examination of male genitalia through dissection is often required to confirm species identity, or to support morphospecies delineation in biodiversity surveys (Boucher and Pollet 2021), but this is not an easy process requiring laborious preparation and expertise. In addition to these challenges, species descriptions are often based on one sex only (more commonly males), making male/ female association difficult, especially when sexually dimorphic species are involved.

DNA barcoding, the sequencing of a short fragment of DNA sequence of the mitochondrial cytochrome c oxidase 1 (CO1) gene, is being increasingly used as an identification tool, especially for very diverse and/or morphologically difficult taxa. DNA barcoding was initially proposed as a tool for the identification of animal species (Hebert et al. 2003), but later found to be useful for many other applications in taxonomy and biodiversity studies including species delineation and biodiversity assessment (Hebert et al. 2016), the discovery of cryptic species, female identification, and male/female association (Janzen et al. 2009; Ekrem et al. 2010; Renaud et al. 2012; DeSalle and Goldstein 2019). The Barcode Index Number (BIN) system (Ratnasingham and Hebert 2013) implemented in the Barcode of Life Data System (BOLD) (Ratnasingham and Hebert 2007) is used to group similar COI sequences into genetic clusters (Molecular Operational Taxonomic Units: MOTUs) that can be used as proxy for species. These genetic clusters are assigned unique identifiers (BINs) and include any barcoded specimens on BOLD (even from unrelated projects) with similar sequences, sometimes providing useful metadata such as locality, elevation, habitat type, sex, picture of the specimen, collection date, sampling technique, and taxonomic assignment if named reference sequences are included in the BIN. This could provide important information for biodiversity inventories and revisionary taxonomic studies (Telfer et al. 2005; Ratnasingham and Hebert 2013).

In the family Agromyzidae, the use of the CO1 gene has been used mainly as a tool to differentiate and identify economically important and invasive species (e.g., Scheffer et al. 2006; Bhuiya et al. 2011; Blacket et al. 2015; Czepak et al. 2018; Firake et al. 2018; Xu et al. 2021), to uncover and identify cryptic species (e.g., Scheffer and Lewis 2006; Scheffer et al. 2014; Weintraub et al. 2017; Mlynarek and Heard 2018), to discover new species (e.g., Scheffer and Wiegmann 2000) and to elucidate Agromyzidae phylogenetic relationships (e.g., Scheffer and Wiegmann 2000; Scheffer et al. 2007; Winkler et al. 2009).

DNA barcoding has rarely been used as a tool for Agromyzidae species identification, morphospecies delineation or gender association in biodiversity surveys, although its use could provide faster and more accurate identification results. Two large biotic surveys occurring in Ontario have used barcoding to provide species identification of thousands of taxa including 21 species (Telfer et al. 2005) and 13 species (deWaard et al. 2018) of Agromyzidae without the expertise of an agromyzid specialist.

A recent and relatively short biotic survey conducted in 2015 at the Mitaraka massif, a mostly unexplored region of French Guiana (Touroult et al. 2018), resulted in 138 agromyzid specimens (43 males; 95 females), delineated into 50 morphospecies (Boucher and Pollet 2021). Based on a combination of external and genitalic characters, male specimens could be delineated into 23 morphospecies, but 69% of the specimens collected were females and morphospecies delineation and male/ female association were highly challenging due to the lack of external diagnostic characters. This problem was especially noticeable for the genera *Melanagromyza* and *Ophiomyia*, the two most abundant and diverse agromyzid genera at Mitaraka (Boucher and Pollet 2021).

Prior to the 2015 Mitaraka expedition, approximately 500 agromyzid species were recorded in the Neotropical region including only four species in French Guiana (*Liriomyzahuidobrensis* (Blanchard), *Liriomyzatrifolii* (Burgess), *Liromyzasativae* Blanchard, *Nemorimyzamaculosa* (Malloch)) (EPPO 2021)). Morphological examination indicated that the Mitaraka agromyzids did not correspond to any of the named species previously recorded for French Guiana (Boucher and Pollet 2021), but some questions remained related to species delineation and identification for the Mitaraka specimens.

The main objective of this study was to investigate whether DNA barcoding could assist with species identification, species delineation, male/ female association, and diversity assessment of the Agromyzidae specimens collected from the Mitaraka Massif (French Guiana) and previously identified as morphospecies (Boucher and Pollet 2021). We also explored if the Barcode Index Number (BIN) system could provide information other than taxonomic assignment (e.g., distribution range, elevation, host plant, etc.) in a region where most of the Agromyzidae fauna is unknown and expected to be undescribed.

## ﻿Materials and methods

Agromyzid specimens were collected in 2015 as part of the Mitaraka expedition, French Guiana (Touroult et al. 2018). The samples were stored in 70% ethanol and subsequently dried using hexamethyldisilazane (HMDS), mounted on cardboard points and identified to morphospecies. A total of 138 specimens representing ten genera and 50 morphospecies were recorded (Boucher and Pollet 2021). Of these, 54 specimens from 5 genera (*Melanagromyza*, *Ophiomyia*, *Nemorimyza*, *Liriomyza*, *Cerodontha*) representing 33 morphospecies of Agromyzidae were selected for DNA barcoding (Tables 1, 2). The selection included 29 specimens of *Melanagromyza* representing all 15 morphospecies, 17 specimens of *Ophiomyia* representing all 14 morphospecies, two specimens of *Nemorimyza*, representing the two morphospecies, five specimens of *Liriomyza* representing one morphospecies, and one specimen of *Cerodontha*, representing the single *Cerodontha* specimen collected from Mitaraka (Boucher and Pollet 2021). In addition to these Mitaraka specimens, one paratype specimen of Cerodontha (Diz) nigrihalterataBoucher (2005) from Costa Rica and housed at the Lyman Entomological Museum was also selected for barcoding for possible comparison with the only *Cerodontha* collected in Mitaraka. The specimens were chosen based on ambiguities and uncertainties that arose during the morphospecies determination (further details below).

**Table 1.** List of Mitaraka specimens sent for barcoding and for which a sequence was retrieved. Includes specimen number for in-text reference, morphospecies name (from Boucher and Pollet 2021), BOLD process ID, BIN assignment, sex, CO1 sequence length, and GenBank accession number. Color text is used when more than one Mitaraka specimen were clustering together in the same BIN (matching color is used in Fig. 1 for easy reference).

Specimen number	Morphospecies	BOLD process ID	BIN assignment (*added for new BIN)	Sex	CO1 Sequence length	GenBank number
1	*Melanagromyza* Mit-1	BUICD1529–19	BOLD:ADX5410*	M	613	OK623732
2	*Melanagromyza* Mit-2	BUICD1440–18	BOLD:ADR6853*	M	658	OK623717
3	*Melanagromyza* Mit-2	BUICD1441–18	BOLD:ADR6853*	M	658	OK623728
4	*Melanagromyza* Mit-2	BUICD1443–18	BOLD:ADR6853*	F	631	OK623740
5	*Melanagromyza* Mit-2	BUICD1444–18	BOLD:ADR6853*	F	658	OK623741
6	*Melanagromyza* Mit-3	BUICD1446–18	BOLD:ADR6852*	M	658	OK623742
7	*Melanagromyza* Mit-4 (previously identified as *M.* Mit-2)	BUICD1445–18	BOLD:ACJ8134	F	658	OK623727
8	*Melanagromyza* Mit-4	BUICD1532–19	BOLD:ACJ8134	F	549	OK623722
9	*Melanagromyza* Mit-4	BUICD1447–18	BOLD:ACJ8134	M	658	OK623729
10	*Melanagromyza* Mit-6	BUICD1534–19	BOLD:ADW8881*	F	602	OK623723
11	*Melanagromyza* Mit-7	BUICD1536–19	BOLD:ADW8881*	F	658	OK623726
12	*Melanagromyza* Mit-9	BUICD1538–19	BOLD:ADB0898	F	658	OK623739
13	*Melanagromyza* Mit-10	BUICD1539–19	BOLD:ADW8248*	F	571	OK623721
14	*Melanagromyza* Mit-10	BUICD1540–19	BOLD:ADW8248*	F	596	OK623733
15	*Melanagromyza* Mit-11	BUICD1541–19	BOLD:ADX5409*	F	555	OK623738
16	*Melanagromyza* Mit-12	BUICD1542–19	BOLD:ADW8247*	M	570	OK623737
17	*Melanagromyza* Mit-12	BUICD1543–19	BOLD:ADW8247*	F	570	OK623735
18	*Melanagromyza* Mit-13	BUICD1544–19	BOLD:ADX3977*	F	658	OK623724
19	*Melanagromyza* Mit-14	BUICD1545–19	BOLD:ADW2860*	F	658	OK623734
20	*Melanagromyza* Mit-15	BUICD1546–19	BOLD:ADX5411*	F	590	OK623736
21	*Ophiomyia* Mit-10	BUICD1558–19	BOLD:ADW4594*	F	564	OK623718
22	*Ophiomyia* Mit-12	BUICD1561–19	Not assigned	F	417	OK623725
23	*Nemorimyza* Mit-1	BUICD1564–19	BOLD:ADW8176*	F	590	OK623720
24	*Nemorimyza* Mit-2	MOBIL8769–18	BOLD:ADB9391	F	600	OK623730
25	*Liriomyza* Mit-1	MOBIL11198–20	Not assigned	F	356	OK623731
26	*Liriomyza* Mit-1	MOBIL11196–20	Not assigned	F	356	OK623719

**Table 2.** Specimens sent for barcoding for which no sequence was retrieved. Includes specimen number for in-text reference, morphospecies name (from Boucher and Pollet 2021), BOLD process ID and sex.

Specimen number	Morphospecies	BOLD process ID	Sex
27	*Melanagromyza* Mit-2	BUICD1442–18	F
28	*Melanagromyza* Mit-4	BUICD1530–19	F
29	*Melanagromyza* Mit-4	BUICD1531–19	F
30	*Melanagromyza* Mit-5	BUICD1533–19	M
31	*Melanagromyza* Mit-6	BUICD1535–19	F
32	*Melanagromyza* Mit-6	Lifescanner Vial ID: BOLD AT1	F
33	*Melanagromyza* Mit-6	Lifescanner Vial ID: BOLD DM0	F
34	*Melanagromyza* Mit-7	Lifescanner Vial ID: BOLD 8E4	F
35	*Melanagromyza* Mit-8	BUICD1537–19	F
36	*Ophiomyia* Mit-1	BUICD1547–19	M
37	*Ophiomyia* Mit-1	BUICD1548–19	M
38	*Ophiomyia* Mit-2	BUICD1549–19	M
39	*Ophiomyia* Mit-3	BUICD1550–19	M
40	*Ophiomyia* Mit-3	BUICD1551–19	F
41	*Ophiomyia* Mit-4	BUICD1552–19	M
42	*Ophiomyia* Mit-5	BUICD1553–19	M
43	*Ophiomyia* Mit 6	BUICD1554–19	F
44	*Ophiomyia* Mit-7	BUICD1555–19	M
45	*Ophiomyia* Mit-8	BUICD1556–19	F
46	*Ophiomyia* Mit-9	BUICD1557–19	F
47	*Ophiomyia* Mit-11	BUICD1559–19	M
48	*Ophiomyia* Mit-12	BUICD1560–19	F
49	*Ophiomyia* Mit-13	BUICD1562–19	F
50	*Ophiomyia* Mit-14	BUICD1563–19	M
51	*Liriomyza* Mit-1	BUICD1449–18	M
52	*Liriomyza* Mit-1	BUICD1448–18	M
53	*Liriomyza* Mit-1	Lifescanner Vial ID: BOLD 5K8	M
54	*Cerodontha* Mit-1	Lifescanner Vial ID BOLD NO6	M
55	*Cerodonthanigrihalterata*	Lifescanner Vial ID BOLD 1N9	F

DNA amplification and Sanger sequencing were performed at the Centre for Biodiversity Genomics (CBG) (previously known as the Canadian Centre for DNA Barcoding (CCDB)) except for specimens #24, 25, 26, 32–34, 51–54 (Tables 1, 2) that were processed through the LifeScanner barcoding service. Tissue samples for DNA extraction, consisting of one or two leg(s) from each specimen, were sent to these institutions following their submission protocols (CBG: http://ccdb.ca/resources/); LifeScanner: http://lifescanner.net/). Primers C_LepFolF/C_LepFolR (Hernández-Triana et al. 2014) were used for DNA amplification of most specimens except the two specimens of *Liriomyza* (#25–26, Table 1) for which primer set MLepF1/C_LepFolR (Hajibabaei et al. 2006) was used. All COI sequences over 400bp were aligned using the Barcode of Life Data System (BOLD) (Ratnasingham and Hebert 2007) and subsequently uploaded in MEGA X (Kumar et al. 2018), where a neighbor-joining (NJ) tree (Saitou and Nei 1987) was built from a distance matrix computed using the Kimura 2-parameter method (Kimura 1980). The NJ tree provides a graphic representation of genetic distance between sequences from a selected dataset. All sequences retrieved from the Mitaraka specimens were compared to the reference sequence libraries of BOLD (using BOLD identification system) and GenBank (using the Basic Local Alignment Search Tool (BLAST)) for a possible match to a named species. All CO1 sequences were deposited in GenBank with accession number listed in Table 1. Collection data, sequences, and specimen photographs are available on the Barcode of Life Data System (BOLD) (dx.doi.org/10.5883/DS-AGROMIT). Specimens from Mitaraka are presently housed in the Lyman Entomological Museum, Ste-Anne-de-Bellevue, QC (**LEMQ**) but will eventually be deposited in the Muséum national d’Histoire naturelle, Paris, France (**MNHN**).

## ﻿Results

**Figure 1.**
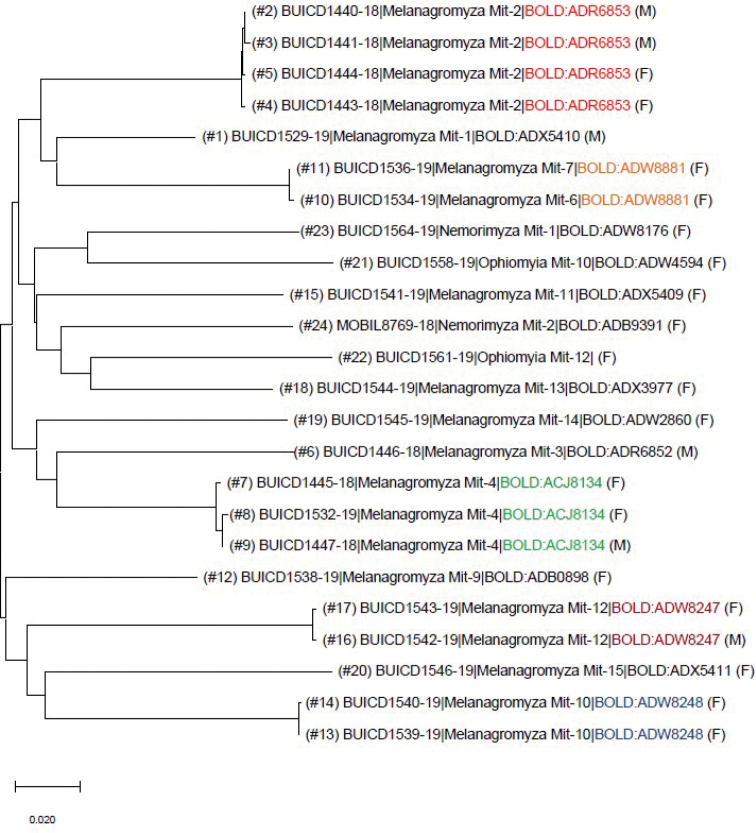
Neighbor-joining tree based on K2P-distance of the 24 specimens of Mitaraka Agromyzidae for which a sequence over 400 bp were retrieved. Information includes specimen number (from Table 1), BOLD process ID, morphospecies name, BIN number and sex. Color text is used when more than one Mitaraka specimen were clustering together in the same BIN.

Amplification success was low (48%), with COI sequences recovered for only 26 of the 54 submitted specimens (Tables 1, 2). Twenty sequences were recovered from *Melanagromyza* specimens, two from *Ophiomyia*, two from *Nemorimyza*, and two short ones of 356 bp from *Liriomyza* (Table 1). None of the COI sequences retrieved from the Mitaraka specimens matched a named species in BOLD or GenBank. In the NJ tree (Fig. 1), the 24 sequences of at least 400 bp representing 17 morphospecies formed 16 distinct clusters with pairwise K2P distances between clusters ranging from 10.7% to 20.9%.

Following the reexamination and subsequent reassignment of specimen #7 (Table 1) to *Melanagromyza* sp. Mit-4, the congruence between morphology and clustering patterns of DNA barcodes was very high, with a single instance of two morphospecies (*Melanagromyza* Mit-6 and *M.* Mit-7) being assigned to the same BIN (BOLD:ADW8881). A total of 15 BINs were assigned to the Mitaraka dataset (Fig. 1, Table 1), all of which were newly created except for BOLD:ACJ8134, BOLD:ADB0898 and BOLD:ADW8248 (Table 1). Even if none of these three BINs were associated to named species in BOLD the presence of sequences from specimens from other localities than Mitaraka provided information on the distribution range of *Melanagromyza* Mit-4, *M.* Mit-9, and *Nemorimyza* Mit-2 (Tables 3, Figs 19, 20).

**Table 3.** Specimen records (public) included in BIN(BOLD:ACJ8134) with associated specimen data.

BOLD identification	BOLD process ID	Sex	CO1 sequence length	Locality/ coordinate/ elevation	Habitat/collecting technique /sampling date
Melanagromyza Mit-4	BUICD1445–18	F	658	Mitaraka, French Guiana, 2.233, -54.463, 471m	Minor inselberg with savane-roche vegetation /6 m Malaise trap/August 2015
Melanagromyza Mit-4	BUICD1532–19	F	549	Mitaraka, French Guiana 2.233, -54.463, 471m	Minor inselberg with savane-roche vegetation /6 m Malaise trap/August 2015
Melanagromyza Mit-4	BUICD1447–18	M	658	Mitaraka, French Guiana 2.233, -54.463, 471m	Minor inselberg with savane-roche vegetation /6 m Malaise trap/August 2015
Agromyzidae	GMAFN352–15	?	633	Reserva El Bagual. Formosa, Argentina -26.3028, -58.815, 57m	Unknown/Malaise trap/November 2013
Agromyzidae	GMCRM972–13	F	658	Area de Conservacion Guanacaste. Guanacaste, Costa Rica 10.8438, -85.6138, 300m	Forest/Malaise trap/May 2012

Detailed results by genus are presented below.

### ﻿*Melanagromyza*

Sequences more than 500 bp were successfully recovered for 20 specimens (69%) belonging to 13 morphospecies and distributed into 12 BINs (Table 1); no sequences were recovered for specimens assigned to *Melanagromyza* Mit-5 and *Melanagromyza* Mit-8 (Table 2).

Sequences from one specimen each of *Melanagromyza* Mit-6 and *Melanagromyza* Mit-7 displayed identical barcodes and were therefore assigned to the same BIN (BOLD:ADW8881) (Table 1; Fig. 1). *Melanagromyza* Mit-7 (2 females) was separated morphologically from *M.* Mit-6 (8 females) by the weaker metallic reflection of the abdomen, ocellar triangle more extended and not as well defined, and body paler. While a BIN merge for *M.* Mit-6 and *M.* Mit-7 could indicate that *Melanagromyza* Mit-6 and Mit-7 are conspecific, it could also represent a case of misidentification for one specimen. Unfortunately, *M.* Mit-6 (specimen #10, Table 1) was lost in the process of tissue sampling, thereby precluding any further morphological comparison with specimen *M.* Mit-7 (specimen #11, Table 1), and no sequences were recovered from the other specimens of *M.* Mit-6 (3 females) and *M.* Mit-7 (1 female) submitted for barcoding (Table 2).

**Figures 2–7.**
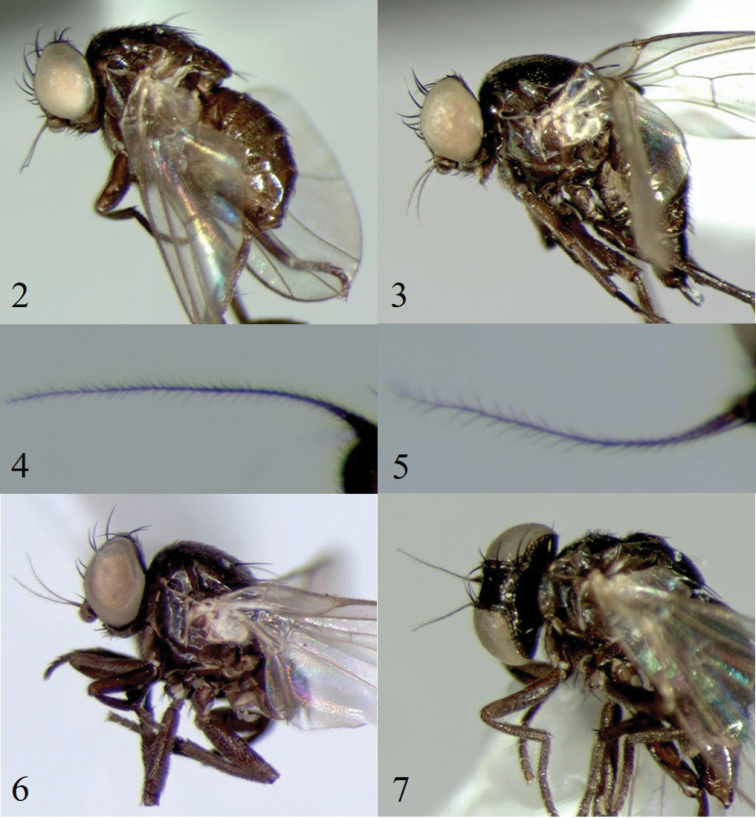
(**2–4**) *Melanagromyza* Mit-2. (**5–7**) *Melanagromyza* Mit-4. **2** specimen BUICD1441–18, lateral view **3** specimen BUICD1444–18, lateral view **4** Arista showing short pubescence **5** Arista showing long pubescence **6** specimen BUIC1447–18, lateral view **7** specimen BUIC1445–18, lateral view.

**Figures 8–12.**
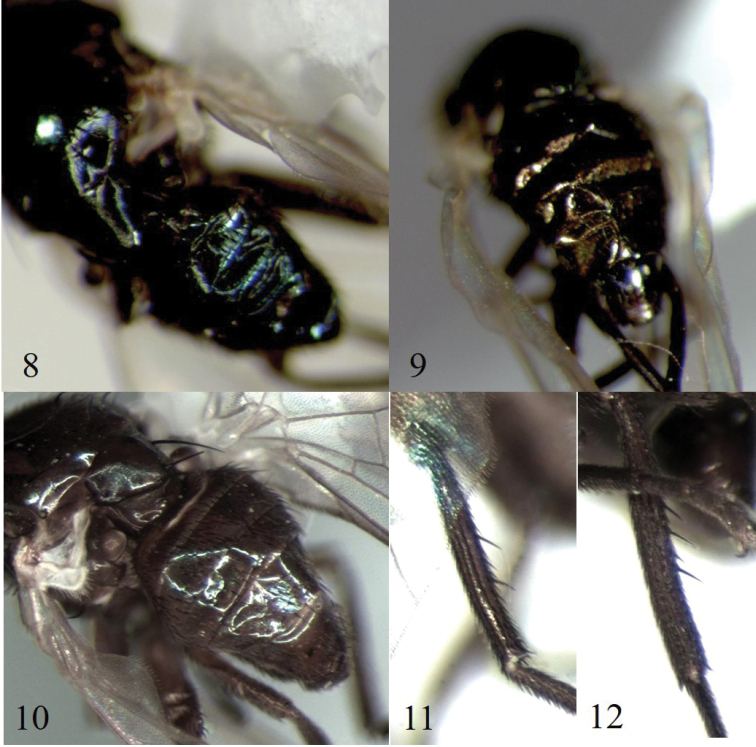
(**8–10**) abdomen (color variation) of *Melanagromyza* Mit-2. **8** specimen BUICD1440–18; **9** specimen BUICD1443–18 **10** specimen BUICD1441–18 (**11, 12**) midtibial bristles (number variation) of *Melanagromyza* Mit-2. **11** specimen BUICD1441–18 **12** specimen BUICD1444–18.

Of the six specimens of *Melanagromyza* Mit-2 submitted for barcoding, only one (#27, Table 2) failed to produce a sequence. Four sequences (2 males and 2 females, #2–5, Table 1) clustered together in BOLD:ADR6853 but one (female #7, Table 1) clustered with material of *Melanagromyza* Mit-4 in BOLD:ACJ8134 (Fig. 1). *Melanagromyza* Mit-2 and *M.* Mit-4 are very similar (Figs 2, 3, 6, 7) except for the shorter pubescence on the arista of *Melanagromyza* Mit-2 (Fig. 4). After re-examination, it was found that specimen #7 (Table 1), previously identified as *Melanagromyza* Mit-2, had long pubescence on the arista matching that of specimens assigned to *Melanagromyza* Mit-4 (Fig. 5). The identification of specimen #7 was therefore updated to *Melanagromyza* Mit-4 (Table 1). *Melanagromyza* Mit-2 was the most common of the Mitaraka Agromyzidae (Boucher and Pollet 2021), but morphological differences were observed between males and some females, including abdomen coloration (Figs 8–10) and number of mid-tibial bristles (Figs 11, 12) which created some uncertainties in gender association. Having sequences from both male and female specimens clustering together in the same BIN (BOLD:ADR6853) with a low sequence divergence, ranging from 0.15 to 0.30% provided additional support for conspecificity.

**Figures 13, 14.**
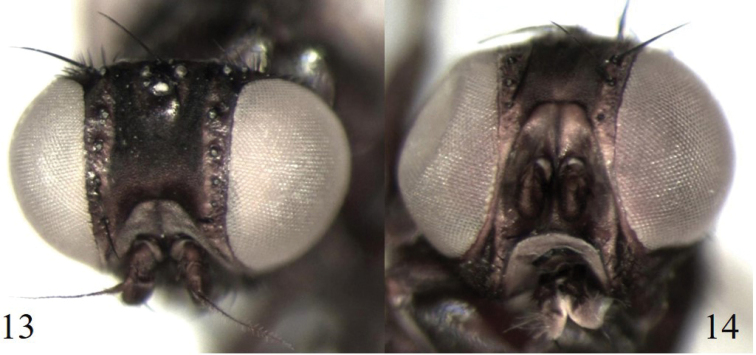
*Melanagromyza* Mit-10. **13** specimen BUICD1539–19, head dorsal view **14** specimen BUICD1540–19, head antero-dorsal view.

Another case of uncertainty in morphospecies determination involved two female specimens (#13–14; Table 1) that were identified as *Melanagromyza* Mit-10 (Boucher and Pollet 2021), although they exhibited slight external differences (Figs 13, 14) including a paler reddish-brown gena, paler lunule and paler anterior orbit for specimen #14. Identical sequences were retrieved for the two specimens and these were assigned to BOLD:ADW8248 (Fig. 1).

Although agromyzid male genitalia are usually species-specific, providing useful characters for species differentiation, it was not the case for males of *Melanagromyza* Mit-3 and *M.* Mit-4 who exhibited very similar genitalia. They were assigned to separate morphospecies based on a few subtle external characters, including a smaller size for *M.* Mit-4 and, in spite of their morphological similarities, material from these morphospecies produced very distinct DNA barcodes with interspecific distances ranging from 11.99% to 12.60%.

**Figure 15–16.**
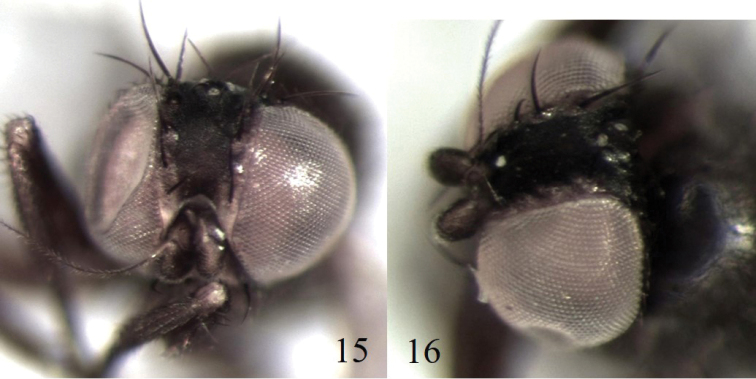
**15***Nemorimyza* Mit-1 BUICD1564–19, head dorsal view **16***Nemorimyza* Mit-2 MOBIL8769–18, head latero-dorsal view.

**Figure 17–18.**
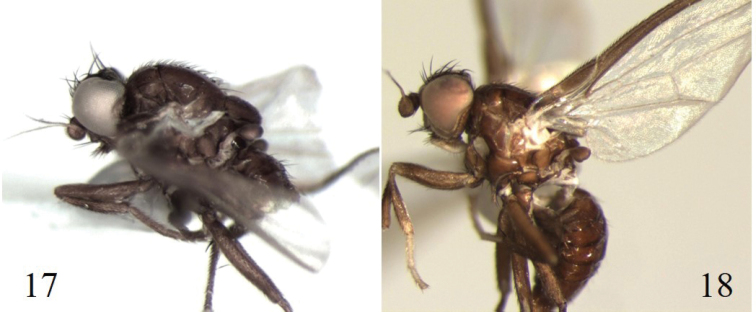
**17***Cerodontha* Mit-1, lateral view **18***Cerodonthanigrihalterata* Boucher, paratype, lateral view.

When sequences were recovered for more than one specimen of a single morphospecies, as seen in *M.* Mit-2, *M.* Mit-4, *M.* Mit-10, and *M.* Mit-12, intraspecific divergences were low, with maximum intraspecific distance (0.37%) recorded in *Melanagromyza* Mit-4 (BIN (BOLD:ACJ8134) (Fig. 1). On the other hand, interspecific distances were high in this genus, ranging from 10.70% between *Melanagromyza* Mit-2 (specimen #4) and *M.* Mit-1 (specimen #1) and 20.90% between *Melanagromyza* Mit-15 (specimen #20) and *Melanagromyza* Mit-6 (specimen #10) (Fig. 1).

**Figure 19.**
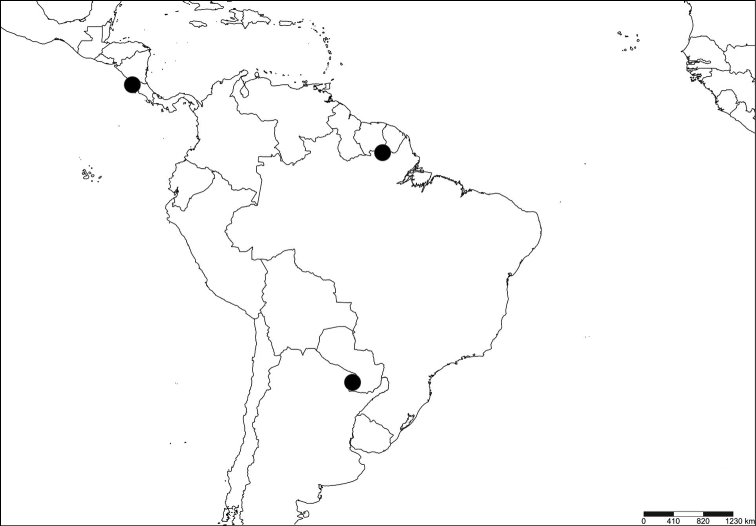
Distribution map for BOLD records for BIN: BOLD:ACJ8134 (*Melanagromyza* Mit-4). Distribution data points include Guanacaste, Costa Rica; Formosa, Argentina and Mitaraka, French Guiana (created with SimpleMappr).

**Figure 20.**
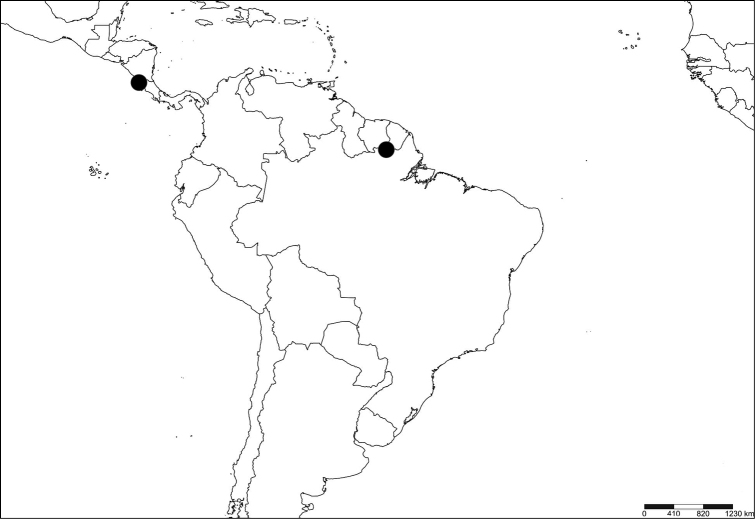
Distribution map for BOLD records for BIN: BOLD:ADB0898 (*Melanagromyza* Mit-9) and BIN (BOLD:ADB9391) (*Nemorimyza* Mit-2). Distribution data points include Guanacaste, Costa Rica and Mitaraka, French Guiana (created with SimpleMappr).

Of the 12 BINs assigned to the Mitaraka *Melanagromyza* specimens, most were new, except BOLD:ACJ8134 and BOLD:ADB0898 (Table 1) that were shared with specimens from other projects. BOLD:ACJ8134 included a total of ten specimens: three specimens from Mitaraka, French Guiana (*Melanagromyza* Mit-4) and seven specimens (two public and five private records) collected in Guanacaste, Costa Rica and Formosa, Argentina (Table 3; Fig. 19). The other shared BIN: BOLD:ADB0898 included the single female specimen of *Melanagromyza* Mit-9 collected at Mitaraka and two specimens (one public record, one private) from Guanacaste, Costa Rica (Table 4; Fig. 20). Surprisingly, *Melanagromyza* Mit-2, the most commonly collected Agromyzidae at Mitaraka (Boucher and Pollet 2021) was attributed a new BIN (BOLD:ADR6853) (Table 1).

**Table 4.** Specimen records (public) included in BIN (BOLD:ADB0898) with associated specimen data.

BOLD identification	BOLD process ID	Sex	CO1 sequence length	Locality /coordinate/elevation	Habitat /collecting technique /sampling date
Melanagromyza Mit-9	BUICD1538–19	F	658	Mitaraka, French Guiana 2.233, -54.463, 471m	Minor inselberg with savane-roche vegetation /6 m Malaise trap/August 2015
Agromyzidae	JICAZ278–16	F	543	Area de Conservacion Guanacaste. Guanacaste, Costa Rica 10.764, -85.335, 828m	Subtropical/tropical moist lowland forest/Malaise trap/March 2014

### ﻿*Ophiomyia*

Amplification success for *Ophiomyia* material was very low, with sequences retrieved from only two of the 17 selected specimens (Tables 1, 2). These sequences (both from females), representing *Ophiomyia* Mit-10 and *Ophiomyia* Mit-12 (Table 1) were separated by an interspecific distance of 18.8% (Fig. 1). The short sequence for *Ophiomyia* Mit-12 (#22, Table 1) did not match an existing BIN and did not meet the 500 bp requirement for erecting a new BIN (Ratnasingham and Hebert 2013). *Ophiomyia* Mit-10 (BUIC-DIP1646) was assigned a new BIN (BOLD:ADW4594) (Table 1).

### ﻿*Nemorimyza*

The five *Nemorimyza* specimens (one male, four females) collected in Mitaraka were originally treated as one morphospecies (*Nemorimyza* Mit-1), until subtle morphological differences were found in two females that were subsequently treated as a distinct morphospecies (*Nemorimyza* Mit-2) (Boucher and Pollet 2021). A sequence over 500 bp was successfully recovered for each of the *Nemorimyza* female specimens representing *Nemorimyza* Mit-1 and *N.* Mit-2 (Table 1). These were assigned to separate BINS, BOLD:ADW8176 and BOLD:ADB9391, and separated by a high interspecific distance of 13.9%. *Nemorimyza* Mit-1 (#23) was assigned a new BIN (BOLD:ADW8176), while *Nemorimyza* Mit-2 (#24) was assigned to BOLD:ADB9391 (Table 1) already containing five other BOLD records (one public) from Guanacaste, Costa Rica (Table 5; Fig. 20).

**Table 5.** Specimen records (public) included in BIN (BOLD:ADB9391) with associated specimen data.

BOLD identification	BOLD process ID	Sex	CO1 sequence length	Locality /coordinate / elevation	Habitat /collecting technique /sampling date
Nemorimyza Mit-2	MOBIL8769–18	F	600	Mitaraka, French Guiana/ 2.233, -54.463/, 471m	Minor inselberg with savane-roche vegetation /6 m Malaise trap/August 2015
Agromyzidae	JCCCY4402–16	F	576	Area de Conservacion Guanacaste. Guanacaste, Costa Rica 10.763, -85.334, 820m	Subtropical/tropical moist lowland forest/ Malaise trap/ November 2014

### ﻿*Liriomyza* Mik

One of the morphospecies (*Liriomyza* Mit-1) collected at Mitaraka was very similar to *Liriomyzasativae*, a species previously recorded in French Guiana, but was treated as distinct based on small male genitalic differences. Of the five male *L.* Mit-1 specimens selected for barcoding, only #25 and #26 produced short sequences of 356 bp (Table 1). These short identical sequences did not match any existing BINs or reference taxon in GenBank and did not meet the 500 bp requirement for erecting a new BIN (Ratnasingham and Hebert 2013). They also had more than 11% genetic distance with reference sequences of *Liriomyzasativae* found in BOLD and GenBank, supporting the assignment of the material to a separate morphospecies.

### ﻿*Cerodontha* Rondani

One morphospecies (*Cerodontha* Mit-1) (Fig. 17) was very similar to Cerodontha (Dizygomyza) nigrihalterata (Fig. 18) a species previously recorded from Costa Rica (Boucher 2005). While a few external characters differentiated *C.* Mit-1 from *C.nigrihalterata*, we could not investigate their genetic differences as no sequences were retrieved for either of the specimens representing these taxa (Table 2).

## ﻿Discussion

There are several possible reasons explaining the low amplification success of the sampled specimens such as the fact that they were not freshly collected and had been kept in 70% ethanol before being dried and mounted, instead of 95% ethanol as recommended for DNA preservation (Nagy 2010). However, most of our specimens were very small (< 2.0 mm) and we suspect that the small amount of tissue submitted for DNA extraction (one or two legs per specimen) may not have been enough.

**Figure 21.**
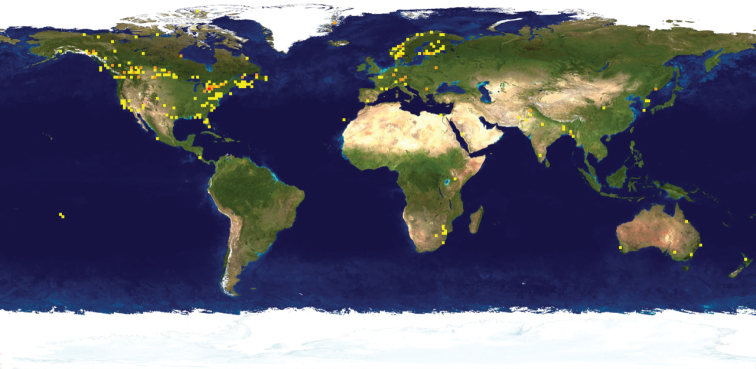
Map of Agromyzidae species occurrence on BOLD. Map generated by BOLD (September 2021).

While DNA barcoding is regularly used as a method of identification for economically important species of Agromyzidae (see introduction), it was not helpful in providing species identification for any of the Mitaraka specimens. This is in part due to the fact that some (if not most) of our material belongs to undescribed taxa. This has been confirmed at least for *Nemorimyza*, where *Nemorimyza* Mit-1 and *N.* Mit-2 do not match any of the five described species (including *N.maculosa*, a species previously reported from French Guiana (EPPO 2021) and with reference sequences available on BOLD from the Nearctic region). Another likely explanation for the absence of a match between our material and reference sequences is the under-representation of identified Neotropical Agromyzidae in BOLD (Fig. 21) and GenBank, making a match unlikely. For example, as of September 2021, there were 540 public records for *Melanagromyza* in BOLD, representing 18 species. More than half (326) of these records (including 319 records from Pakistan) represent *Melanagromyzaobtusa* (Malloch), a well-known economically important species recently reported in the Americas, including Colombia (Martinez-Alava et al. 2016). Of the remaining 17 species, only one, *Melanagromyzaminimoides* Spencer is from the Neotropical region and none of the barcoded Mitaraka specimens matched that species.

As for *Liriomyza*, most reference sequences in BOLD belong to economically important species and this barcode library is important to facilitate the identification of the most important agromyzid pests. As of September 2021, there were 3411 public records of *Liriomyza* in BOLD representing 49 species. More than half (1803) of these records belong to four agricultural pests: *L.sativae* (677 records); *L.trifolii* (668 records); *L.brassicae* (Riley) (339 records) and *L.huidobrensis* (119 records), all recorded from the Neotropical region. Other than these four species, no other named Neotropical species of *Liriomyza* have been barcoded, except for five specimens of *L.nigra* Spencer (with short sequences of 307 bp) belonging to a private project managed by the first author. The short sequence retrieved for *Liriomyza* Mit-1 did not match those of any *Liriomyza* species found in BOLD. Further investigation will be required to confirm the identity of *Liriomyza* Mit-1. The genus *Liriomyza* is the most diverse agromyzid genus in the Neotropical region with approximately 105 species known. Species level identification is difficult due to the lack of recent keys to the Neotropical species and the fact that some species that have been described based on female specimens only (e.g., *L.mikaniovora* Spencer from Venezuela; *L.pagana* (Malloch) from Argentina and *L.quiquevittata* Sasakawa from Chile).

**Figure 22.**
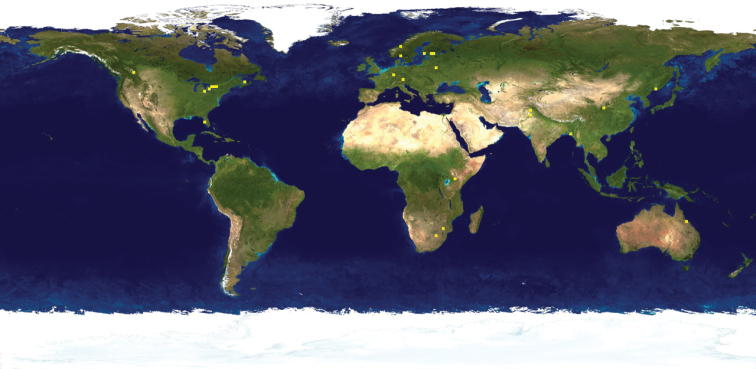
Map of *Melanagromyza* species occurrence on BOLD. Map generated by BOLD (September 2021).

Although DNA barcoding and the BIN system were not useful to assign names to any of our morphospecies, they did provide information relevant to the taxonomy and diversity of the Mitaraka agromyzid fauna. They allowed us to flag and reassess the identification of some specimens (see results under *Melanagromyza*) and assisted with male/female associations. Due to the importance of male genitalic character for species recognition in agromyzids, females are often left unidentified in taxonomic and faunistic studies (Černý and Bächli 2018; Eiseman and Lonsdale 2018), excluded from type series because of uncertainties in gender association (eg: *Calycomyzaaddita*Spencer (1983)) or left undescribed or unnamed in the absence of conspecific male (e.g., *Liriomyza* sp. B (Boucher and Wheeler 2014); *Japanagromyza* “female 1” (Lonsdale 2013)). Females can be particularly abundant in biodiversity surveys, especially when Malaise traps are used (Scheirs et al. 1997). This was the case for the Mitaraka survey where 95 females and 43 males were collected (Boucher and Pollet 2021). In the present work, DNA barcoding supported the male/ female conspecificity of specimens assigned to three *Melanagromyza* morphospecies (*M.* Mit-2, *M.* Mit-4, *M.* Mit-12). Furthermore, the high sequence divergence measured between branches or clusters of barcoded morphospecies (Fig. 1) supported almost all the morphospecies assignments even when these were erected only based on female material. The sequencing of additional material will be needed to further investigate the grouping of *Melanagromyza* Mit-6 and *M.* Mit-7 in the same BIN (BOLD:ADW8881) due to the accidental destruction of the only specimen of *M.* Mit-6 with a DNA barcode.

Very little data was available on the agromyzid fauna of French Guiana before the 2015 Mitaraka survey. The high congruence between DNA barcodes/ BIN assignments and morphology presented here suggests that DNA barcoding is an effective approach to estimate the Agromyzidae species diversity of Mitaraka and beyond, especially when females are abundant in samples. Additional studies will be necessary to further evaluate the robustness of the approach since it is widely recognized that levels of congruence between species limits and DNA barcodes/ BINS vary according to the study group. While causes such as hybridization and incomplete lineage sorting (Funk and Omland 2003) are most commonly evoked, simple errors in morphology-based identification can also account for mismatches, especially in the case of morphologically challenging taxa such as agromyzid flies. An approach combining multiple data sources such as morphology, DNA sequences, and life history traits such host plants should therefore be favored whenever possible.

The genus *Melanagromyza* was the most diverse at Mitaraka with 15 morphospecies (Boucher and Pollet 2021). This diversity resulting from a short survey in a single locality of French Guiana was surprisingly high when compared to known diversity of *Melanagromyza* in different Neotropical countries such as Brazil (19 species), Venezuela (20 species), or Colombia (14 species). The diversity of *Melanagromyza* from the Mitaraka survey could even be greater considering that 70% of the identified specimens were not sequenced and could include cryptic species that failed to be differentiated morphologically. We therefore suspect that much is left to be discovered about the agromyzid fauna of French Guiana and the Neotropical region in general.

We also found that the Barcode Index Number (BIN) system, along with the metadata associated with each barcoded specimen in BOLD, provided important insight into the distribution pattern, habitats, and elevation preference of some species (Tables 3–5), in addition to allowing researchers to locate material easily for revisionary taxonomic studies.

Considering the difficulty associated with species-level identification of Neotropical Agromyzidae and the risks associated with the postal transport of type material, a reference library of DNA barcodes for named species of Neotropical Agromyzidae (including sequences from type material whenever possible) would not only help with identification but also reduce taxonomic errors that may lead to long lists of synonyms such as seen for several species of economic importance such as *L.sativae* and *L.brassicae*.

This study has contributed a total of 23 new barcode-compliant CO1 sequences (more than 500 bp), of Neotropical Agromyzidae, distributed into 15 BINs (including 12 unique BINs). Although these sequences lack species-level determination, they set a stronger base for future taxonomic work and facilitate the discovery of conspecific supplementary material for morphological studies.
